# The Number of Scholarly Documents on the Public Web

**DOI:** 10.1371/journal.pone.0093949

**Published:** 2014-05-09

**Authors:** Madian Khabsa, C. Lee Giles

**Affiliations:** 1 Computer Science and Engineering, The Pennsylvania State University, University Park, Pennsylvania, United States of America; 2 Information Sciences and Technology, The Pennsylvania State University, University Park, Pennsylvania, United States of America; Wayne State University, United States of America

## Abstract

The number of scholarly documents available on the web is estimated using capture/recapture methods by studying the coverage of two major academic search engines: Google Scholar and Microsoft Academic Search. Our estimates show that at least 114 million English-language scholarly documents are accessible on the web, of which Google Scholar has nearly 100 million. Of these, we estimate that at least 27 million (24%) are freely available since they do not require a subscription or payment of any kind. In addition, at a finer scale, we also estimate the number of scholarly documents on the web for fifteen fields: Agricultural Science, Arts and Humanities, Biology, Chemistry, Computer Science, Economics and Business, Engineering, Environmental Sciences, Geosciences, Material Science, Mathematics, Medicine, Physics, Social Sciences, and Multidisciplinary, as defined by Microsoft Academic Search. In addition, we show that among these fields the percentage of documents defined as freely available varies significantly, i.e., from 12 to 50%.

## Introduction

Many researchers and academics are concerned about the extent to which academic and scientific documents are available on the web, as well as their ability to access them. For convenience, we will refer to all academic and scientific documents as “scholarly”. By scholarly documents, we mean journal and conference papers, dissertations and masters theses, books, technical reports and working papers. Patents are excluded.

The web has become a standard resource for such documents because individual authors, academic and research publishers, and repositories have made their documents available online, with some open to the public and others limited to subscribers.

Numerous databases and search engines such as Google Scholar and CiteSeer track scholarly documents and thus facilitate research. However, the coverage of some of these search engines and databases is unknown. An important question that a scholar or researcher might ask is whether a single search engine or database is sufficient to obtain comprehensive results in a particular field. For example, *Web of Science* reported that as of January 2013 it comprises more than 49.4 million records [1], and Microsoft Academic Search (MAS) stated that it covers 48.7 million documents[2]. However the size of Google Scholar is unknown despite studies that have tried to determine the extent to which Scholar's citations overlap with those of other citation indices [3], [4]. Relatively smaller digital libraries and databases, such as CiteSeer and PubMed, tend to focus on documents from certain fields, most of which are also indexed by large search engines such as Google Scholar and MAS. Bjork et al. [5] estimated the number of published papers in 2006 to be roughly 1.35 million, whereas a similar estimate for 2011 put the number at 1.8 million [6]. But despite the availability of per year estimates, researchers have yet to provide an estimate of the total number of published scholarly documents.

Estimating the number of scholarly documents available on the web is quite different from estimating the size of the web itself, and thus presents different challenges. Studies that offer estimates of the size of the web such as Lawrence and Giles [7], [8], Bharat and Broader [9], or Dobra and Fienberg [10] can not be used to estimate the number of scholarly documents on the web for many reasons. For example, search engines are no longer receptive to automated requests for fear of denial of service attacks or reverse engineering of their ranking function. Checking that a document indexed by search engine *A* is also available in the index of search engine *B* is nontrivial. To estimate the size of the web, one strategy would be to check whether a particular URL is available in both engines. However, in the case of scholarly documents the search engines might not have obtained their copies from the same location since the same document might be available at different URLs. Therefore, it is necessary to explore the content of the document and not just the location from which it was obtained. Even when a search engine returns the location of a certain document, it could be that the publisher offers full access to subscribers only and has a limit on the number of downloads allowed per day, thus making automated methods impractical. Finally, many publishers restrict access for many web crawlers.

## Estimating the Number of Scholarly Documents on the Web

To estimate the number of scholarly documents on the web, we use the relative size of two major academic search engines: Google Scholar (Scholar) and Microsoft Academic Search (MAS). We note that our estimates are limited to English documents only. We used the option offered by Google Scholar of filtering results by language, whereas for MAS we ran a language detection algorithm on the title of each document. Only those identified as English were used. Our approach can be described as follows. Assuming that each academic search engine would sample the web independently for papers, then each index would contain a subset of available documents. Next, we considered each search engine to be a random capture of the document population at a certain time. Using the intersection of these two captures, we estimate the entire size of the population. However, since obtaining the database of both academic search engines was not feasible, we approximated the overlap by randomly sampling from each search engine and then determining the size of overlap in the random sample. The simplest approach for sampling from two search engines is to send queries to each and then measure the overlap of the results. This approach was used by Lawrence and Giles [7], [8] and by Bharat and Broader [9]. However, it is known to suffer from many biases and statistical dependencies. To mitigate the effect of bias and dependence and to obtain a selection that was as random as possible, we sampled from each academic search engine with the following methodology: if we choose a random paper *p* that is in the database of an academic search engine, then the set of papers *S* that cite *p* is a random collection from this search engine. If we collect the set of papers citing *p* from both Google Scholar and MAS, then the overlap between these two is an estimate of the overlap between the two search engines. This method provides a good estimate of the coverage of each search engine because when an academic search engine builds its database by indexing a new document, it has no knowledge of the incoming citations to this document. Therefore, the search engine has to obtain all the available manuscripts and analyze them in order to determine whether there are any citations to a target paper. In contrast to references, which the search engine can extract from the document and try to obtain a copy of each referenced item, incoming citations are not embedded with a document. Hence, to build a complete citation network, it is necessary for a search engine to obtain all the available scholarly documents. The more documents the search engine obtains, the larger its citation network.

Based on the methodology described, we chose 10 documents from each of the fifteen fields specified by Microsoft Academic Search: Agriculture Science, Arts and Humanities, Biology, Chemistry, Computer Science, Economics and Business, Engineering, Environmental Sciences, Geosciences, Material Science, Mathematics, Medicine, Physics, Social Sciences, and Multidisciplinary. The list of papers used as queries for which we retrieved the collection of incoming citations was randomly chosen from the most cited documents in each field. Special care was taken in regard to choosing documents because search engines impose a limit on the maximum number of retrievable results. Therefore, the chosen documents each had fewer than 1,000 citations in Scholar and likewise fewer than 1,000 citations in MAS.

The experiments were performed during the period of January 10–12, 2013 by, (1) sending 150 requests to each search engine requesting the list of incoming citations to each paper such that each request corresponds to one paper, and (2) storing the returned metadata about each citation which included the document's title, list of authors, number of citations, year of publications, and the venue of publication (if available). Overall, we obtained 41,778 citations from MAS and 86,870 citations from Google Scholar. Matching the citations across results from different sources (Scholar and MAS) cannot be achieved solely on the basis of verbatim matching of title and authors. The reason is that academic search engines obtain their metadata in different ways. For example, a publisher might provide some or all of the metadata. Alternatively, the metadata of the document might be automatically extracted from a downloaded document from the web. In the latter case, errors are inevitably introduced in the extraction stage resulting in noisy metadata. Though we have no way of establishing whether a certain paper's metadata was provided by a publisher or automatically extracted, we found evidence that the results are mix of both cases. Another issue was that Scholar and MAS differed occasionally in terms of their respective result encoding, especially with regard to Latin letters. Therefore, the records returned by MAS and Scholar for a given paper were matched as follows. To match the Scholar citation collection 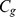 with the MAS citation collection 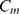, for the same paper, we first matched each paper in 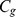 with its counterpart in 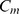 such that the papers' titles were exact textual match. Later, we constructed shingles of size two for all the titles in both 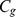 and 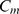. The collection of size two shingles for a title is the set containing every two continuous words appearing in that title [11]. For example, the size two shingles for the sentence: “A Brief History of Time” would be 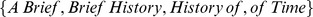. Given the set of shingles 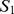 for a paper in 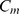, and the set 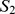 for a paper appearing in 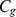, we computed Jaccard similarity between 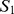 and 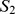 as follows:
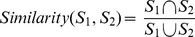
We computed the Jaccard similarity between every pair of documents appearing in 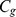 and 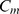, and considered a pair 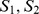 to be a match when their similarity was above a certain threshold. Based on our experiments with different values of the threshold for accuracy, we empirically selected 0.50. After matching the collections 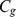 and 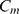 as described, we manually evaluated the matched records individually for mistakes. We found mistakes in less than 2% of the matched records, and all false negatives and positives were corrected. Overall, more than 4,000 record pairs were manually inspected.

**Figure 1:**
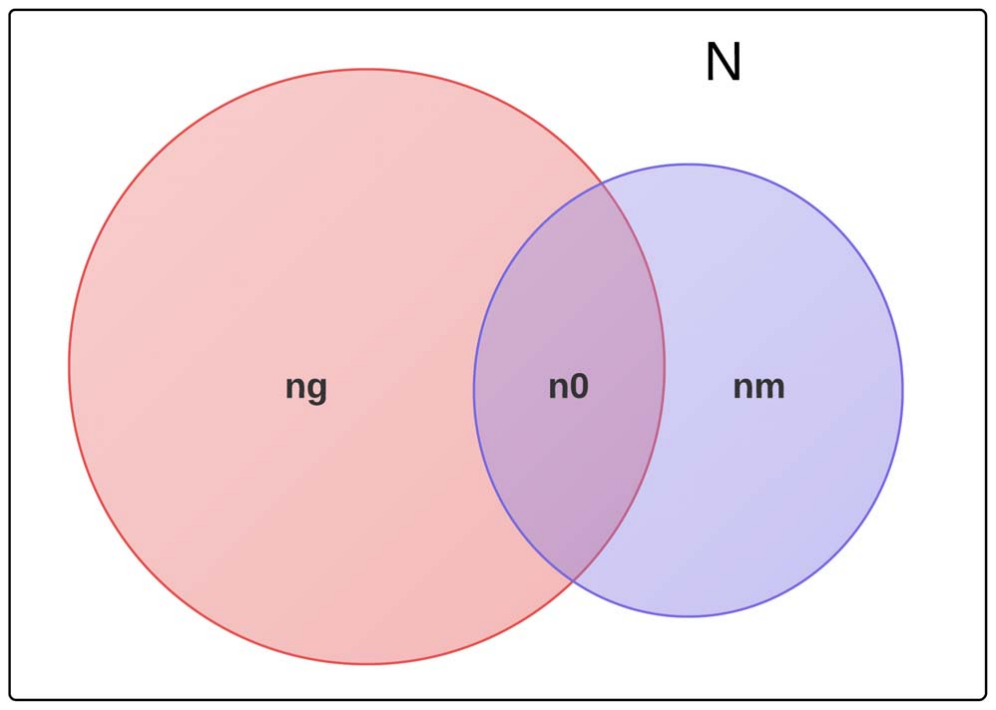
To estimate the number of scientific documents on the web, 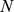, let 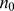 equal the number of citations found in both Scholar and MAS for a collection of papers, and let 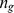 be the number of citations reported by Scholar. Then 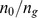 is an estimate of 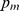,the fraction of documents indexed by MAS. The total number of documents N would be 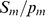 where 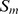 is the size of MAS.

We computed the overlap between the results for all the 150 query documents, and measured the total number of unique documents that cited the query documents. The overall size of scholarly documents on the web can be estimated using capture/recapture (refer to Appendix S1 for an introduction to capture/recapture). Assuming that the total number of documents on the web is 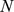, and each search engine samples the web independently, then the quantity 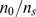 where 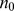 is the number of documents returned by both Scholar and MAS, and 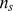 is the number of documents returned by Scholar is an estimate of the fraction of scholarly documents, 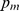, indexed by MAS. Then, the number of documents on the web 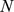 can be estimated as 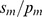 where 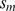 is the number of documents indexed by MAS. These variables are illustrated in Figure 1. At the time of this study, 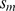 was listed as 48,774,763 by MAS. However, according to our analysis 98% of the returned papers from MAS were found to be in English. Therefore, in our estimates we used 0.98 * 48774764 = 47799267 as an estimate of the number of English papers in MAS, 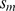. Next, 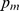 was estimated to be 0.418, yielding an estimate size of 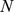, the total number of documents on the web, of 114,000,000.

We argue that this estimate is a lower bound of the number of scholarly documents on the web because the likelihood that a document is in an academic search engine given that it was found in another academic search engine, is larger than the likelihood that any given document is indexed by an academic search engine. Although we designed our experiments to mitigate any possible statistical dependence by relying on citations instead of query results, the experiments do introduce a bias against documents with more than 1,000 citations. Search engines impose a restriction on the number of retrievable results for all type of queries, unless an Application Programmable Interface (API) is provided. Hence, any study based on sampling from a search engine, regardless of the approach, would encounter this bias. For our study it is relevant to note that Google Scholar at this time does not provide an API.

**Figure 2:**
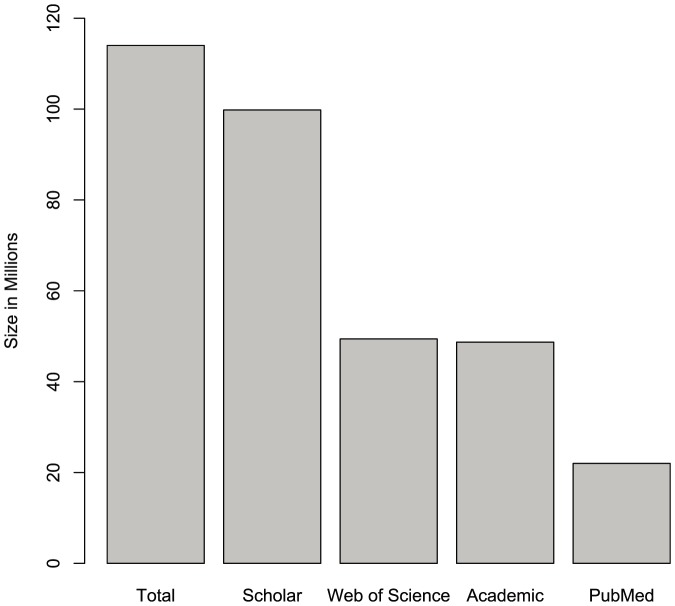
Relative number of documents by scholarly search engines and databases. Total and Google Scholar are estimates.

Using the statistics calculated above, we estimated Google Scholar to have 99.3 million documents, which is, approximately, 87% of the total number of scholarly documents found on the web. This percentage is close to the 86% reported by Norris, Oppenheim and Rowland [12] when they tested the coverage of Google and Google Scholar for finding Open Access documents. With this estimated size, Google Scholar is more than twice as large as the nearest alternative, as MAS and Web of Science are both reported to have fewer than 50 million records. However, we estimate that Scholar fails to index 13% of all web accessible documents. This implies that it is necessary to search across multiple search engines in order to retrieve a comprehensive list of results. The relative size of each database/search engine is depicted in Figure 2.

## Field Level Analysis

In addition to computing statistics about the total number of scholarly documents on the web, we can reinterpret the experiments at the field-scale, making it possible to obtain estimates of the size of each of the fifteen scholarly fields defined in MAS. To obtain these estimates, we assumed that a paper and its citations belonged to the same field. Though this assumption does not always hold, we assumed that it would be a good approximation to the number of citations within a discipline. We also noted that it is possible for some papers to be classified into multiple fields especially in closely related fields, e.g. engineering and mathematics. Nevertheless, as the number of citations grew for a given paper, we anticipated more papers from the same field would cite it.

Using the classification provided by MAS, and the number of papers reported in each field, we used the 10 queries in the experiments for each field to compute the overlap between Scholar and MAS in that particular field. Table 1 reports the estimate of the total number of available documents using the procedure described above (method #1 in the table).

**Table 1:** The estimated number of documents on the web for each field.

Discipline	Size in MAS	Estimate of Size #1	Estimate of Size #2
Agriculture Science	447,134	1,088,711	1,026,904
Arts & Humanities	1,373,959	5,286,355	3,155,485
Biology	4,135,959	8,019,640	9,498,798
Chemistry	4,428,253	10,704,454	10,170,091
Computer Science	3,555,837	6,912,148	8,166,468
Economics & Business	1,019,038	2,733,855	2,340,360
Engineering	3,683,363	7,947,425	8,459,349
Environmental Sciences	461,653	975,211	1,060,249
Geosciences	1,306,307	2,302,957	3,000,113
Material Science	913,853	3,062,641	2,098,789
Mathematics	1,207,412	2,634,321	2,772,987
Medicine	12,056,840	24,652,433	27,690,190
Physics	5,012,733	13,033,269	11,512,430
Social Science	1,928,477	6,072,285	4,429,012
Multidisciplinary	9,648,534	25,798,026	22,159,184
Total Sum		121,223,731	117,540,415

**Figure 3:**
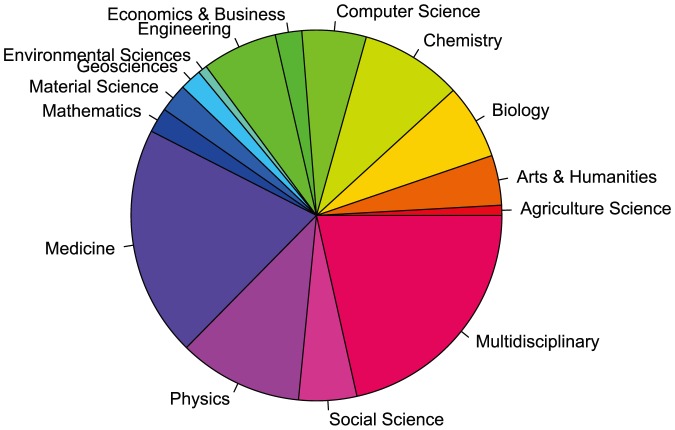
The relative number of documents on the web for each of the 15 fields as defined by MAS.

The sum of the individual field estimates yields a total of 121 million, (last row of Table 1) which is close to the 114 million estimate obtained earlier for the total number of documents across all fields. This supports our assumption that the per field estimate is fairly accurate, and is not much affected by cross field references or the chance of assigning a paper to multiple fields. Hence, the numbers estimated in Table 1 are indicative of the actual size of each field. The relative size of each field is shown in the pie chart in Figure 3. In addition to computing a capture/recapture estimate for the size of each field, we report on another method for this estimate. In this approach, each field's size is computed as the percentage of total available documents on the web based on our previous estimate of 114 million for the total number of scholarly documents. The percentage is obtained by computing the field's percentage size in MAS. For example, MAS is reported to have 4,135,959 documents in biology. Therefore the percentage of biology to the total number of scholarly documents is 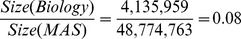. Thus, with this method (method #2), the estimate of the total number of documents in biology is 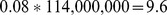 million. We notice here that the assumption of citations belonging to the same field is under sampling certain fields, while over sampling others. However, it is quite close to the percentage-based estimate in many fields.

Another interesting estimate is the percentage of scholarly documents on the web that are freely available, i.e. can be accessed without paying a fee or needing a subscription. We used Google Scholar to estimate this percentage because Scholar provides a direct link to the publicly available document next to each search result where a link is available. Note that there is no easy way to distinguish between publisher's links and public links in MAS. As our estimate found that Scholar contains only 87% of the available scholarly documents on the web, our estimate of the percentage of public documents is limited to the coverage of Scholar. However, this is still a good indicator of the relative availability of publicly available documents. To estimate the percentage of publicly available documents for each field, we randomly sampled 100 documents from MAS belonging to each field such that each document had at least one citation. We imposed a citation limit to filter out documents that are collected by MAS that were not real scholarly documents (although it is rare to find such documents, they nevertheless exist). Then, each of the 100 documents was searched on Google Scholar to establish whether the document was freely available on any site. The percentage of freely available documents for each field is reported in Table 2. In the last two columns, we multiply the estimate of the percentage of freely available documents by the size estimate of the field in Table 1 (method #1), resulting in the total number of freely available documents in that field.

**Table 2:** The percentage of publicly available scholarly documents found in Google Scholar.

Field	% of Public	95% CI	Estimate of Size	95% Lower Bound
Agriculture Science	12	±6.3	130,645	72,446
Arts & Humanities	24	±8.3	1,268,725	897,331
Biology	25	±8.4	2,004,910	1,433,666
Chemistry	22	±8.1	2,354,979	1,625,540
Computer Science	50	±9.8	3,456,074	2,887,549
Economics & Business	42	±9.6	1,148,219	926,256
Engineering	12	±6.3	953,691	528,852
Environmental Sciences	29	±8.8	282,811	210,017
Geosciences	35	±9.3	806,034	625,341
Material Science	12	±6.3	367,516	203,799
Mathematics	27	±8.7	711,266	518,878
Medicine	26	±8.5	6,409,632	4,630,828
Physics	35	±9.3	4,561,644	3,539,034
Social Science	19	±7.6	1,153,734	761,868
Multidisciplinary	43	±9.7	11,093,151	8,992,160
Total			36,703,036	27,853,573

The 95% one sided lower bound confidence interval for the estimated number of freely available documents is 27.8 million, which accounts for roughly 24% of the total estimate of scholarly documents. This estimate is a weighted average of the one sided 95% lower bound confidence interval of the percentage of freely available documents in each field multiplied by the respective estimated field size. The lower end of the one sided confidence interval is for the proportion size, and is computed as:
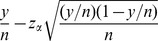
where 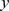 is the number of publicly found documents, 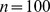, and 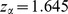 is the standard normal distribution at 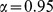 [13].

It would be interesting, however, to determine the quality of these freely available documents. It is also worth pointing out that this estimate of 24% for the percentage of publicly accessible scholarly documents is a bit higher than the 15–20% documents estimated to be self-archived [14], [15].

Note here that our sampling is uniform, because we retrieved the document IDs of all the documents in each given field from MAS, then uniformly chose 100 that conformed to the citation sampling restriction. To the best of our knowledge, this is the only uniform sampling method for estimating the percentage of freely available scholarly documents. The numbers reported in Table 2 differ from other recent estimates in regard to the number of documents available on the web as open access, e.g. Bjork et. al. [16]. We believe this difference arises from the sources from which they sampled. For the other recent estimate researchers considered only journals over the period of one year, whereas our definition of scholarly documents is not limited to journals and sampling was cumulative, i.e. not limited to any time period. Compared to other kinds of publications, journal publications are more likely to be indexed by databases such as Web of Science [5]. However, other documents such as conference proceedings and technical reports, though influential may not be indexed by Web of Science. As an example, the famous PageRank paper [17], which presents the seminal algorithm for Google ranking was published as a technical report. Therefore, Web of Science does not index it.

In summary, the lower bound estimate of the number of scholarly documents, published in English, available on the web is roughly 114 million, of which Google Scholar covers nearly 87%, approximately 100 million documents. Therefore, it would be useful for researchers to consider as a standard practice querying multiple databases and academic search engines in order to gain the most comprehensive result for their query. Also, we estimate that almost 1 in 4 of web accessible scholarly documents are freely and publicly available. Our estimates for specific academic fields differs significantly, such that some fields have 4 times greater percentage of freely available documents than others.

## Supporting Information

Appendix S1 **Appendix providing size estimates using other methods.**
(PDF)
