# Supplementary material for: The Number of Scholarly Documents on the Public Web
Source: PLoS One. 2014 May 9;9(5):e93949. doi: 10.1371/journal.pone.0093949 (PMC4015892; doi:10.1371/journal.pone.0093949)
Supplement: Appendix S1 — Appendix providing size estimates using other methods. (PDF) [file pone.0093949.s001.pdf]

Supplementary Statistics for The Number of Scholarly Documents on the Public Web

Madian Khabsa[1], C. Lee Giles[1,2,*]
1 Computer Science and Engineering, The Pennsylvania State University, University Park, PA, USA
2 Information Sciences and Technology, The Pennsylvania State University, University Park, PA, USA
∗ E-mail: Corresponding giles@ist.psu.edu

Abstract

This appendix reports how we estimated the number of scholarly documents on the web using another method based on Poisson regression capture/recapture. The estimates obtained using this method are similar to the estimates reported in the paper and provide validation for our approach.

Additional Estimates

The approach we used to estimate the total number of scholarly documents on the web is usually reported in the literature as the Lincoln-Petersen method [1, 2]. It also happens to be the maximum likelihood estimator of a population size when the captures are modeled as a hypergeometric distribution [3]. These methods are based on the assumption that the population size does not change between captures, and that the probability of capturing an object in the later captures does not change after the first capture. Assume that n_1 items were captured, then released after being labeled. A second capture results in n_2 items, of which m are labeled from the first capture. Then, the estimate of the population size N is given by

$$\hat{N} = \frac{n_1 n_2}{m}$$

In our experiment for estimating the total number of scholarly documents, the assumption of a closed population size is not completely preserved because search engines constantly add new documents. But since we confined the time window of the experiment to two days, and given that the number of citations grows slowly (unlike news articles), we argue that the assumption of a closed population is a reasonable approximation. However, as we previously point out, the conditional probability of search engine B capturing a document previously captured by a search engine A is larger than or equal to the probability of capturing a document by B. This is primarily due to the tendency of web crawlers to index pages that are connected to other pages, i.e. more popular pages (a similar argument was made by Lawrence and Giles [4]). Although in practice the capture probabilities differ between captures, we argue that this approach produces a good estimate of the total population size, and a good approximation of the total number of scholarly documents on the web.

To test the validity of this assumption, we used an estimate that allows the probability of capturing items across different occasions to vary. As such, the probability of capturing an item i on the second capture can differ from the probability of capturing i at the first capture. Next, with *Rcapture* [5], Poisson regression was used to estimate the parameters of the capture/recapture model [6–8]. Using Poisson regression, the estimate of the population size as computed by *Rcapture*, while allowing for variability with regard to capture probability across time, would put the percentage of scholarly documents covered by MAS at 0.418, hence estimating the number of scholarly documents on the web to be 114 million. This is exactly the Maximum Likelihood Estimator value obtained using the Lincoln-Petersen method. And it is also the approach with the lowest Akaike Information Criterion (AIC) [9], a measure of relative goodness of fit. Note that the lower the value of the AIC, in this case 41, the better fit of the data.

A 95% confidence interval of the population size results in a coverage percentage for MAS in range of (0.416,0.419). However, if the probability by which items are captured is assumed to be the same across the captures, then the Poisson regression model would estimate a population size that would put the coverage percentage of MAS at 0.366. Therefore, the total number of scholarly documents on the web would be estimated as 130 million. This approach, on the other hand, has a high AIC of 41914 which indicates poor goodness of fit compared with the varying capture probabilities model.

In conclusion, our experiments are supported by two methods for estimating the document population. As both methods obtain the same estimate, we reported in the paper the simpler method based on the maximum likelihood estimator, Lincoln-Petersen.

References

1. Lincoln FC (1930) Calculating waterfowl abundance on the basis of banding returns. US Department of Agriculture Circular 118.

2. Petersen C (1896) The yearly immigration of young plaice into the limfjord from the german sea. Report of the Danish Biological Station 6: 1–48.

3. Sheldon R, et al. (2009) A First Course In Probability, 8/E. Pearson Education.

4. Lawrence S, Giles C (1998) Searching the world wide web. Science 280: 98–100.

5. Baillargeon S, Rivest LP (2007) Rcapture: loglinear models for capture-recapture in r. Journal of Statistical Software 19: 1–31.

6. Cormack RM (1989) Log-linear models for capture-recapture. Biometrics : 395–413.

7. Cormack R, Jupp P (1991) Inference for poisson and multinomial models for capture-recapture experiments. Biometrika 78: 911–916.

8. Cormack R (1992) Interval estimation for mark-recapture studies of closed populations. Biometrics : 567–576.

9. Akaike H (1974) A new look at the statistical model identification. Automatic Control, IEEE Transactions on 19: 716–723.
